# Transcriptional repression of the M channel subunit Kv7.2 in chronic nerve injury

**DOI:** 10.1016/j.pain.2010.12.028

**Published:** 2011-04

**Authors:** Kirstin Rose, Lezanne Ooi, Carine Dalle, Brian Robertson, Ian C. Wood, Nikita Gamper

**Affiliations:** a Institute of Membrane and Systems Biology, Faculty of Biological Science, University of Leeds, Leeds, UK; b Pain TA, Pfizer Global Research and Development, Sandwich, UK

**Keywords:** KCNQ, M current, REST, Sensory neuron, DRG, Neuropathic pain

## Abstract

Neuropathic pain is a severe health problem for which there is a lack of effective therapy. A frequent underlying condition of neuropathic pain is a sustained overexcitability of pain-sensing (nociceptive) sensory fibres. Therefore, the identification of mechanisms for such abnormal neuronal excitability is of utmost importance for understanding neuropathic pain. Despite much effort, an inclusive model explaining peripheral overexcitability is missing. We investigated transcriptional regulation of the *Kcnq2* gene, which encodes the Kv7.2 subunit of membrane potential-stabilizing M channel, in peripheral sensory neurons in a model of neuropathic pain—partial sciatic nerve ligation (PSNL). We show that *Kcnq2* is the major *Kcnq* gene transcript in dorsal root ganglion (DRG); immunostaining and patch-clamp recordings from acute ganglionic slices verified functional expression of Kv7.2 in small-diameter nociceptive DRG neurons. Neuropathic injury induced substantial downregulation of Kv7.2 expression. Levels of repressor element 1–silencing transcription factor (REST), which is known to suppress *Kcnq2* expression, were upregulated in response to neuropathic injury identifying the likely mechanism of *Kcnq2* regulation. Behavioural experiments demonstrated that neuropathic hyperalgesia following PSNL developed faster than the downregulation of *Kcnq2* expression could be detected, suggesting that this transcriptional mechanism may contribute to the maintenance rather than the initiation of neuropathic pain. Importantly, the decrease in the peripheral M channel abundance could be functionally compensated by peripherally applied M channel opener flupirtine, which alleviated neuropathic hyperalgesia. Our work suggests a novel mechanism for neuropathic overexcitability and brings focus on M channels and REST as peripheral targets for the treatment of neuropathic pain.

Neuropathic injury induces transcriptional downregulation of the *Kcnq2* potassium channel gene by the transcriptional suppressor repressor element 1–silencing transcription factor; this mechanism contributes to peripheral sensitization of the afferent fibres.

## 1 Introduction

Nerve injury or degeneration often results in the development of neuropathic pain. This type of pain constitutes an enormous health problem because it is particularly difficult to treat with conventional analgesics. Often neuropathic pain is caused by the unprovoked and uncontrolled activity of peripheral nerve fibres [11]; the search for the mechanisms of such peripheral overexcitability is ongoing, but a clear picture remains elusive.

When peripheral sensory fibres are severed, the proximal part of the nerve forms a terminal swelling or endbulb, which may then form a neuroma [11,14]. Both the neuroma and the cell bodies of damaged nerves generate anomalous ectopic firing [2,15,27,51], with inflammation [38], sympathetic sprouting [9,30] and changes in the expression of various genes related to neuronal excitability [53] being identified as likely contributing factors.

One of the major mechanisms controlling tonic excitability of mammalian neurons is M-type K^+^ current [7] conducted by Kv7 channels (encoded by the *Kcnq1-5* genes). In neurons most M channels are formed by homomeric or heteromeric association of Kv7.2, Kv7.3, and Kv7.5 [10,52]. Because of their distinctive biophysical properties (slow activation and deactivation, no inactivation, and a threshold for activation below −60 mV), M channel activity maintains strong control over neuronal excitability. Genetic deficiency or acute inhibition of M channels in neurons leads to overexcitability (eg, seizures), whereas M channel openers have an antiexcitatory effect [10]. Recently functional M channels were identified in sensory neurons [25,26,36]. Moreover, it has been demonstrated that acute inhibition of M channels in nociceptors causes depolarization, increases excitability, and produces nocifensive behaviour in rats [25,26].

Recently we demonstrated that *Kcnq* genes have functional repressor element 1 (RE1) binding sites that are able to recruit repressor element 1–silencing transcription factor (REST, also called neuron-restrictive silencer factor, NRSF) leading to inhibition of *Kcnq* transcription [32]. Thus, overexpression of REST in dorsal root ganglia (DRG) neurons robustly suppressed M current density and increased tonic excitability of these neurons [32]. Baseline REST expression in neurons is low, but it was shown to increase greatly after inflammation [32] or after the neuropathic injury [49]. Thus, we hypothesized that transcriptional downregulation of *Kcnq* gene expression by REST may contribute to neuropathic hyperexcitability of DRG neurons. To test this concept we characterised expression of *Kcnq* genes in DRG and studied transcriptional regulation of the major *Kcnq* transcript, *Kcnq2*, in an animal model of neuropathic pain. We show that the expression of *Kcnq2* is strongly suppressed in DRG after neuropathic injury, an effect most likely mediated by REST as its nuclear expression in neurons was upregulated. Because M channels maintain neuronal resting membrane potential, *Kcnq2* downregulation would contribute to ectopic activity of neuropathic fibres. Accordingly, application of the M channel opener flupirtine directly to the site of injury reduced neuropathic hyperalgesia. Our findings describe a novel mechanism contributing to peripheral sensitization after nerve injury, reinforce Kv7 channels as a peripheral drug target for treatment of pain, and identify REST as a potential novel target in pain therapeutics.

## 2 Materials and methods

### 2.1 Acute DRG slice preparation

DRGs were sliced in accordance with Scholz et al. [42]. Briefly, DRGs were embedded in liquid 2% w/v agar and sectioned (all steps on ice) with a vibroslicer (Leica VT1000S, Leica Microsystems, Nussloch GmbH, Germany) at 190 μm thick in artificial cerebrospinal fluid solution (in mM; 124 NaCl, 26 NaHCO_3_, 10 glucose, 3 KCl, 2 MgSO_4_, 2.5 NaH_2_PO_4_, 2 CaCl_2_) that was bubbled with carbogen. No enzymatic treatment was used during the acute slice preparation of DRG and during the subsequent patch clamp recording.

### 2.2 Electrophysiology

An amphotericin B perforated patch was used for patch clamp recordings as described elsewhere [25]. The intracellular pipette solution contained (in mM): 140 KCl, 1 MgCl_2_, 10 HEPES, 10 EGTA, 1 CaCl_2_; artificial cerebrospinal fluid solution (see earlier) was used as extracellular solution. The recordings were made using EPC-10 amplifier and Patchmaster 2.2 software (both from HEKA Elektronik, Lambrecht, Germany). To evaluate the amplitude of M current, DRG neurons were held at −30 mV, and 1-second hyperpolarising steps to −60 mV followed by a 1-second step to −30 mV, were applied every 3 seconds.

### 2.3 Animals

Adult (150 to 200 g) male Wistar rats (bred in-house) were used for this study. Rats were housed in the University of Leeds Animal facility in groups of 4 on a 12:12 light–dark schedule. Food and water were available ad libitum. After delivery, rats were maintained within the in-house animal facility for at least 2 days before surgery or behavioural testing. All experimental procedures on animals followed the guidelines and recommendations of the UK Home Office and were in accordance with the regulations of the UK Animals (Scientific procedures) Act 1986.

### 2.4 Partial sciatic nerve ligation (PSNL) surgery

Partial ligation of the left sciatic nerve was performed as previously described [43]. Briefly, male Wistar rats (150 to 200 g) were anesthetised with 2% v/v isoflurane; the left sciatic nerve was exposed at mid-thigh level and cleared of surrounding connective tissues. A 6-0 Prolene suture (Ethicon Ltd. Edinburgh, UK) was inserted into the nerve with a 3/8 curved, reversed-cutting mini-needle, and tightly ligated so that the dorsal third/half of the nerve was held within the ligature. A small cut was made approximately 5 mm below the ligature. The wound was then closed using 2 to 5 skin clips (Reflex, Harward Apparatus, Kent, UK). In sham-operated rats, the left sciatic nerve was exposed but untouched.

### 2.5 Peri-sciatic nerve injection

Lidocaine (0.65% v/v) and M channel modulators flupirtine (50 μM) and XE991 (10 μM) were injected in a volume of 200 μL to the area surrounding the sciatic nerve to directly deliver drugs to the neuroma site. The concentration of lidocaine used in this study was based on Ma et al. [29]; concentrations of the XE991 and flupirtine were based on Wang et al. [52] and Luisiet al. [28], respectively. Rats were briefly anaesthetised with isoflurane (until tail pinch reflex was absent but eye blink reflex remained). The needle was inserted into the left thigh until it made contact with the thigh bone and then brought back few millimetres, and solution was injected gradually over 3 seconds.

### 2.6 Thermal hyperalgesia (Hargreaves test)

Change in withdrawal latency to noxious heat was recorded using the Hargreaves plantar method [19] (UgoBasile, Stoelting, Italy). Rats were acclimated for at least 20 minutes before the test. The heat source was placed directly under the plantar surface of the hind paw, and the latency of paw withdrawal (in seconds) was measured with automated digital timer. Pre-surgery thermal latency values were recorded between 1 and 4 days before PSNL injury or sham surgery. During these baseline recordings the infrared intensity was adjusted with a constant voltage power supply to obtain a baseline response time of approximately 12 seconds, and the same settings were used throughout the whole series. Both ipsilateral and contralateral hind paw withdrawal latencies were recorded 15 and 30 days after surgery.

### 2.7 Mechanical hyperalgesia (Randall-Selitto algesymeter paw-withdrawal test)

The nociceptive flexion reflex was quantified with an Ugo Basile Analgesymeter 37215, which applies a linearly increasing mechanical force to the dorsum of the rat hind paw [37]. The mechanical nociceptive threshold was defined as the force in grams at which the rat withdrew its hind paw. Rats were acclimated for at least 20 minutes before the test. Mechanical thresholds were recorded as the mean of 3 measurements at 10-minute intervals. Baseline mechanical thresholds were established between 0 and 4 days before surgery. Behavioural testing resumed 15 and 30 days after surgery.

### 2.8 DRG and sciatic nerve removal

Rats were killed by schedule 1 technique in accordance with Home Office guidance. A laminectomy of the lumbar region was carried out. Scissors were used to cut the lamina and body of the spinal vertebrates until the left and right sides of the column were separated. The spinal cord and meninges were then carefully removed to allow removal of DRG. After removal of DRG, any attached nerves were cut and then DRGs were prepared for reverse transcriptase–polymerase chain reaction (RT-PCR), Western blot, or immunohistochemistry as described later. To excise sciatic nerve, the skin overlying the thigh was removed and muscles overlying the nerve were separated using large scissors at the mid-thigh level. Approximately a 1 cm length of sciatic nerve was cut at mid-thigh height and used for immunohistochemical studies.

### 2.9 Quantitative RT-PCR

Total RNA was extracted from homogenised L4/L5 DRG using Quiazollysis reagent (Qiagen, Crawley, UK). Contaminating DNA was removed using a DNA-free kit (Applied Biosystems, Warrington, UK). RNA was reverse transcribed using M-MLV reverse transcriptase (Promega, Southampton, UK) per manufacturer’s protocol. Resulting cDNA was purified using a QIAquick PCR purification kit (Qiagen, Crawley, UK). Quantitative RT-PCR was performed on a MyIQ Real-time RT-PCR detection system (BioRAD, Hemel Hempstead, UK). For each primer set, water and no RT RNA samples were used in place of a template as negative controls. The following primers were used: *Kcnq2* sense 5′-CCGGCAGAACTCAGAAGAAG; *Kcnq2* antisense 5′-TTTGAGGCCAGGGGTAAGAT; *Rest* sense 5′-CGAACTCACACAGGAGAACG; *Rest* antisense 5′-GAGGCCACATAATTGCACTG; *U6* sense 5′-CTCGCTTCGGCAGCACA; *U6* antisense 5′-AACGCTTCACGAATTTGCGT. SYBR green was used as a reporter. The cycle at which fluorescence increases above background (threshold cycle, Ct) was measured during the exponential phase of the PCR. Expression levels of genes were normalized to those of *U6*.

### 2.10 Western blot

Protocol used for nuclear-enriched preparation of L4/L5 DRG neurons was adapted from Berti-Mattera et al. [5]. Briefly, DRGs were homogenised on ice in hypotonic buffer (in mM; 10 HEPES, pH 7.9, 10 KCl, 10 EDTA, 1 DTT, 0.3% Tirton-X v/v, 57 mOsm, supplemented with protease inhibitor tablet [Roche, West Sussex, UK]). Nuclei were pelleted by centrifugation at 9000*g* for 5 minutes at 4°C. The resulting supernatant (cytosolic fraction) was removed and stored at −80°C. The pellet was resuspended in high-salt buffer (in mM; 20 HEPES, pH 7.9, 400 NaCl, 1 EDTA, 10% glycerol v/v, 1 DTT, 2.5 Osm, supplemented with protease inhibitor tablet) and placed on a rocker at 4°C for 2 hours. Cell debris was pelleted by centrifugation at 9000*g* for 5 minutes at 4°C. The supernatant was the nuclear-enriched fraction. For total protein extraction of kidney and forebrain, rough tissue homogenate was placed into a glass–glass homogeniser and 50 μL RIPA buffer (50 Tris–HCl, pH 7.4, 150 NaCl, 1 EDTA, 1% v/v Triton-X v/v, 1% v/v sodium deoxycholate, 0.1% w/v SDS, supplemented with protease inhibitor tablets) was added before homogenising samples. Samples were freeze-thawed between dry ice and 37°C water bath and centrifuged 9000*g* for 10 minutes at 4°C. The protein samples were then analysed using SDS-PAGE Western blot using standard protocol. REST, β-actin, and Nup62 were detected using rabbit anti-REST (Upstate/Millipore, Chicago, IL; 1:1000) mouse anti-β-actin, (Sigma, York, UK 1:10000) and mouse anti-Nup62 (ProteinTech Group, Inc. Manchetser, UK; 1:1000) antibodies, respectively. Nikon Elements AR 3.0 (Nikon UK Ltd., Kensington upon Thames, UK) software was used to quantify signal intensities. The REST intensity was normalized to that of Nup62 for each protein sample.

### 2.11 Immunohistochemistry

The standard immunohistochemical protocol requires fixation of the tissue section, which provides mechanical stability and antigen retention within the tissue preparation. However, in the present study fixation of tissue sample caused a loss of antigen specificity and nonspecific binding of the Kv7.2 antibody (gift of Prof. Mark S Shapiro, UTHSCSA, USA; the antibody has been characterized in Roche et al. [39]). Thus a fresh-frozen tissue preparation was used. The L4 DRGs or sciatic nerve were removed and embedded in Tissue-Tek (optimal cutting temperature [OCT] solution, Leica Microsystems, Milton Keynes, UK) on dry ice. Cryostat sections (10- to 25-μm slices) were thaw-mounted on slides coated with poly-L-lysine and gelatine, and stored at −20°C. Once mounted, slices were incubated in phosphate-buffered saline (PBS) for 5 minutes and then in PBS, 0.1% Triton-X for a further 10 minutes at room temperature. Slices were then rinsed in PBS for 3 × 5 minutes at room temperature and incubated in primary antibodies (dilutions listed in Table 1) overnight at 4°C. After primary antibody incubation, slices were washed in PBS for 3 × 5 minutes at room temperature. Slices were then incubated with appropriate secondary antibody (dilutions listed in Table 1) for 3 hours at room temperature. For co-labeling studies, incubation with neurochemical marker always preceded Kv7.2 labeling. Slides were then mounted using Vectashield plus DAPI (Vector Laboratories, Burlingame, CA). For each staining procedure, PBS without primary antibody was used as a negative control where cells were detected by the presence of DAPI staining. When tissue samples from naive, sham-operated, and PSNL animals were prepared for the purpose of the comparison, each tissue sample was sliced consecutively into 8 slices and each slice was mounted onto the separate glass slide; this was done to enable mounting of individual tissue slices from the contralateral and ipsilateral sides of the sham and PSNL animals onto the same slide (so that the staining and imaging conditions were identical for each series). A similar slicing protocol was used for colocalization studies so that the repeats of a tissue sample on the same slide were separated by at least 80 μm to avoid labelling of same cells for averaging numbers per DRG. Confocal microscopy was performed with a Zeiss LSM 510 Meta microscope (Carl Zeiss, Welwyn Gardens, Herts, UK); all settings were adjusted at the beginning of the session and were not altered throughout the session. Images were analysed using Nikon Elements AR 3.0 (NIS) software.

### 2.12 Statistical analysis

All data were assessed for normality using the Kolmogorov–Smirnov normality test. Where data were not normally distributed, Kruskal–Wallis one-way analysis of variance (ANOVA) on Ranks was used with Dunn’s multiple comparison procedure. Where data were normally distributed, one-way ANOVA with Holm–Sidak multiple comparison procedure or Student’s *t*-test were used as appropriate. Statistical analyses were performed with SigmaStat 3.1 software package (Systat Software Inc., Richmond, CA).

## 3 Results

### 3.1 Expression of functional Kv7 channels in DRG neurons

M channels in DRG neurons are formed by Kv7.2, Kv7.3, and Kv7.5 subunits [36]; thus, we first analysed relative expression of *Kcnq2, Kcng3,* and *Kcnq5* mRNAs in whole DRG lysates using quantitative RT-PCR. Relative levels of *Kcnq2*, *Kcnq3*, and *Kcnq5* mRNA (normalised to the level of housekeeping gene *U6*) were 0.31 ± 0.05 (n = 6), 0.07 ± 0.01 (n = 6), and 0.002 ± 0.001 (n = 7), respectively. Thus, relative *Kcnq2* mRNA expression was approximately 4-fold greater than that of *Kcnq3* (*P* ⩽ .05) and 180-fold greater than that of *Kcnq5* (*P* ⩽ .01); we therefore focused our study on *Kcnq2*.

**Fig. 1:**
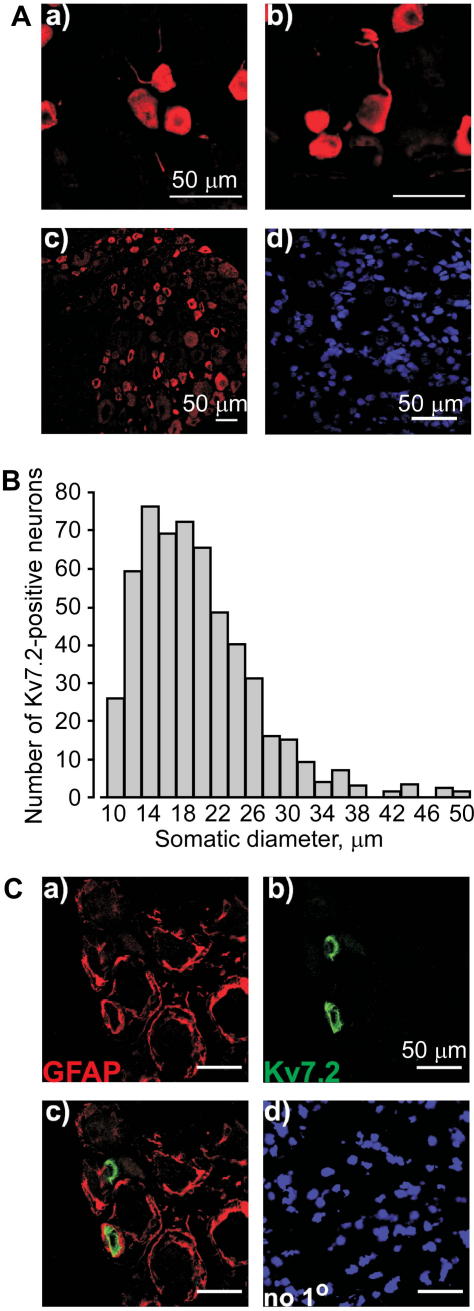
Expression of Kv7.2 in DRG neurons. (A) Confocal micrographs at 40× (a and b) and 10× (c and d) magnification of DRG sections stained with anti-Kv7.2 antibody (a–c), or no-primary control (d). Nuclei were stained with DAPI (d). Scale bars = 50 μm. (B) The cell diameter of Kv7.2-positive neurons within L4 DRG was measured to the nearest micrometer and is presented as a frequency distribution. (C) Kv7.2 immunoreactivity was not detectable within satellite glial cells (SGC) of DRG. Confocal micrographs for immunostaining with antibodies against the glial marker GFAP (a); Kv7.2 (b); co-staining (c) and no-primary control (d). Scale bars = 50 μm.

**Fig. 2:**
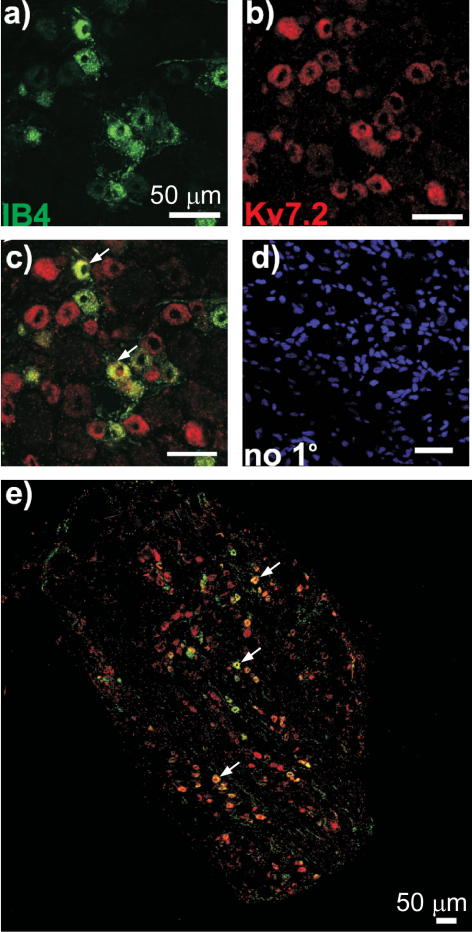
Colocalization of Kv7.2 and IB4 in small DRG neurons. Confocal micrographs for immunostaining with antibodies against the nonpeptidergic nociceptive neuron marker IB4 (a); Kv7.2 (b); co-staining (c and e), and no-primary control (d). Scale bars = 50 μm. Examples of co-staining are indicated with arrows.

**Fig. 3:**
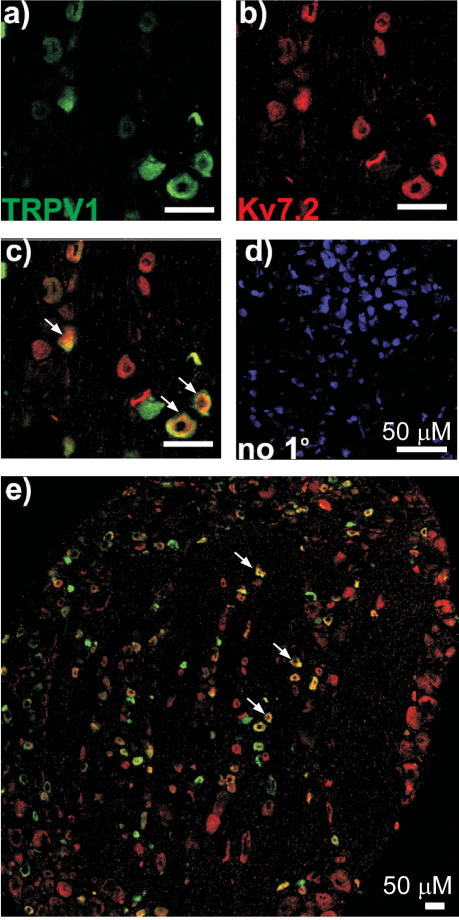
Colocalization of Kv7.2 and TRPV1 in small DRG neurons. Confocal micrographs for immunostaining with antibodies against the nociceptive neuron marker TRPV1 (a); Kv7.2 immunostaining (b), co-staining (c and e), and for no-primary control (d). Scale bars = 50 μm. Examples of co-staining are indicated with arrows.

**Fig. 4:**
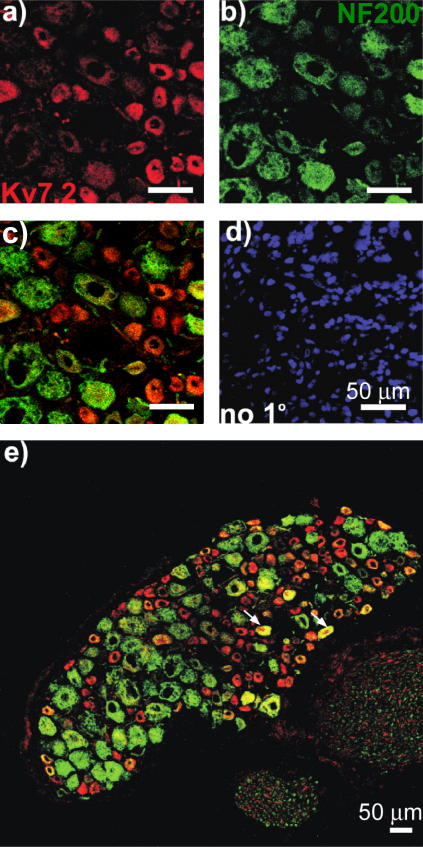
Colocalization of Kv7.2 and NF200 in DRG neurons. Confocal micrographs for immunostaining with antibodies against Kv7.2 (a); large mechanosensitive DRG neuron marker NF200 (b), co-staining (c and e), and for no-primary control (d). Scale bars = 50 μm. Examples of co-staining are indicated with arrows.

**Fig. 5:**
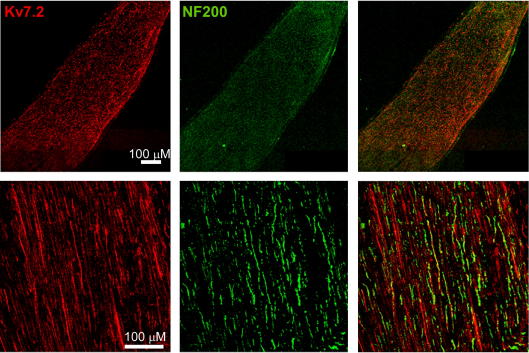
Expression of Kv7.2 within the sciatic nerve. Confocal micrographs for immunostaining of rat sciatic nerve with antibodies against Kv7.2 (a); large mechanosensitive DRG neuron marker NF200 (b), co-staining (c and e) and no-primary control (d). Scale bars = 50 μm.

Immunostaining of DRG sections (Fig. 1) demonstrated robust expression of Kv7.2 in neurons of predominantly small diameter (20.2 ± 0.3 μm, n = 547) (Fig. 1A and B). Both cell bodies and axonal sprouts of DRG neurons displayed Kv7.2 expression (Fig. 1A). Kv7.2 immunoreactivity did not colocalize with glial marker glial fibrillary acidic protein (GFAP) (Fig. 1C). In contrast, Kv7.2 staining was colocalized with the markers of nociceptive neurons IB4 (Fig. 2) and TRPV1 (Fig. 3). Of the Kv7.2-positive neurons, 32.8 ± 2.6% were IB4-positive (829 neurons/4 rats) and 47.0 ± 4.9% were TRPV1-positive (687 neurons/4 rats). In contrast, only 12.4% of Kv7.2-positive neurons were positive for a marker of large, mechanosensitive neurons, NF200 (Fig. 4). Thus, Kv7.2 is predominantly expressed in small-diameter nociceptive neurons within rat DRG. Staining of the sciatic nerve also revealed abundant Kv7.2 immunoreactivity (Fig. 5), which as in the case for DRG cell bodies showed poor colocalization with NF200, suggesting there is limited Kv7.2 expression within the larger-diameter DRG neuron fibres [41].

**Fig. 6:**
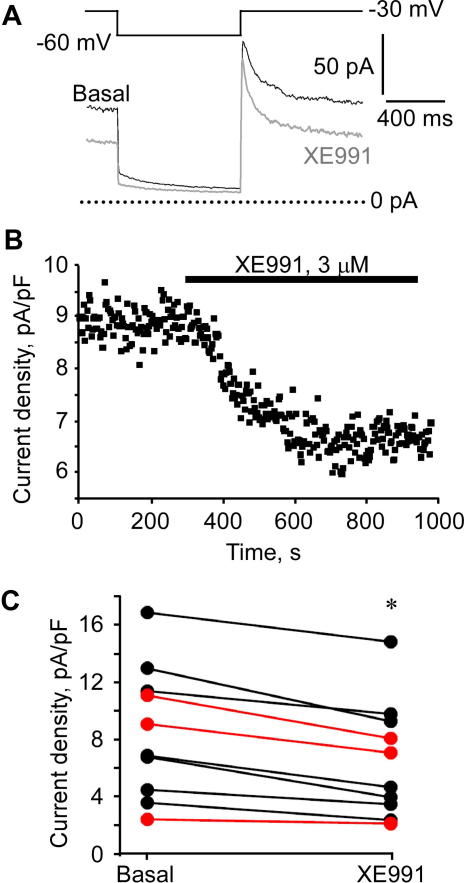
M current recording from acute DRG slices. (A) Perforated patch recording of M-like current from a small-diameter neuron in an acute DRG slice in the absence (basal) or presence (XE991) of 3 μM XE991; voltage protocol is depicted earlier. (B) Time course of M current inhibition by 3 μM XE991 (black bar) during a DRG slice recording. (C) Comparison of M current density in individual neurons from P7 (black) and adult (red) rat DRG slices recorded at −30 mV in the absence (basal) or presence of XE991 (^∗^*P* < .05).

To verify that *Kcnq2* expression results in the formation of functional M channels in native DRG tissue, we performed perforated patch-clamp recordings from acute L4/L5 DRG slices from newborn (P7) or adult rats (Fig. 6). Slow-inactivating M-like currents that were sensitive to specific M channel blocker XE991 (3 μM) were recorded from small (cell capacitance of 16 ± 2 pF) DRG neurons. The satellite glial cells that ensheath adult DRG are very rigid, making recording from adult DRG neurons from acute slices technically difficult (to our knowledge no adult DRG slice recordings of M current have been reported to date), thus we only obtained 3 such recordings (from more than 10 rats) although these recordings were consistent with those from newborn DRGs (Fig. 6C, red). In neurons from newborn rats, 3 μM XE991 inhibited 26.6% ± 4.1% (2.1 ± 0.4 pA/pF) of total outward current at −30 mV (8.9 ± 1.8 pA/pF, n = 7) and 10 μM XE991 inhibited 60.0% ± 15.9% of outward current at−30 mV (n = 3). Because high concentrations (100s μM) of XE991 can block HERG channels [13], we applied XE991 (3 μM) in the presence of the HERG blocker terfenadine (1 μM). When HERG channels were blocked with terfenadine, XE991 still inhibited the outward current by 2.5 ± 1.1 pA/pF (n = 4; data not shown), suggesting that the M-like current was not HERG but indeed M current. The deactivating current at −60 mV best fit to a double exponential function that gave fast and slow time constants of 18 ± 4 ms and 249 ± 45 ms, respectively (within the range of values previously reported for Kv7.2 [35]). All recorded neurons responded to 1 μM capsaicin (not shown). These data represent the first electrophysiological characterization of M current in acute DRG tissue; taken together, evidence presented in this section suggests that: (1) *Kcnq2* is the predominant *Kcnq* transcript in DRG, (2) Kv7.2 forms functional M channels in DRG, and (3) many Kv7.2-expressing neurons are nociceptors.

### 3.2 Transcriptional downregulation of Kcnq2 expression after neuropathic injury

**Fig. 7:**
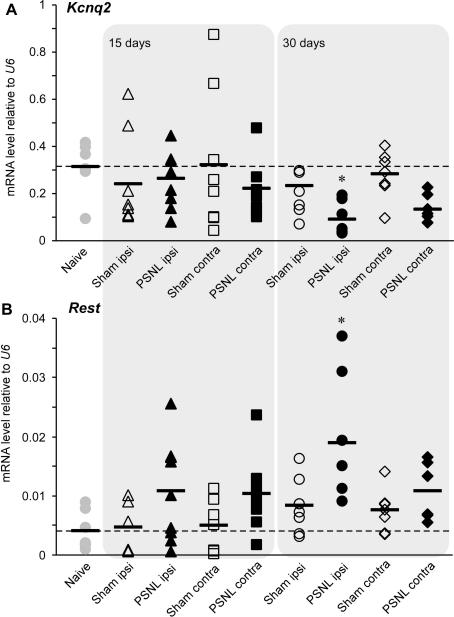
Reciprocal changes in *Kcnq2* and *Rest* mRNA after peripheral nerve injury. Relative *Kcnq2* (A) and *Rest* (B) mRNA levels in naive, sham-operated, and PSNL animals 15 and 30 days after surgery. All data are normalised to levels of the house keeping gene, *U6.* Differences were analysed using Kruskal–Wallis one-way analysis of variance on ranks, multiple comparisons versus control group (Dunn’s method; ^∗^*P* < .05, n ⩾ 6).

The experiments in this section were based on the following previous findings: (1) neuropathic injury is often characterized by the sustained overexcitability of peripheral nociceptors [11]; (2) acute inhibition of M current in nociceptive neurons by inflammatory mediators produces short-term excitability and acute pain [25,26]; (3) neuropathic injury induces a large-scale remodelling of nociceptive neurons driven by long-term changes in gene expression [53]. Thus we reasoned that because acute M channel inhibition in DRG causes short-term excitability, long-term hyperexcitability observed in some neuropathic pain conditions can be in part caused by the downregulation of M channel expression. Therefore we used quantitative RT-PCR to test whether the expression of *Kcnq2* is regulated in an animal model of neuropathic injury — PSNL. To minimize the contribution of acute trauma and postoperational inflammation, samples were taken 15 and 30 days postsurgery (see later for the behavioural manifestations of neuropathic trauma at these time intervals). Fifteen days postsurgery, relative *Kcnq2* mRNA expression levels (normalized to *U6*) in ipsilateral and contralateral L4/L5 DRG from sham and PSNL rats were not significantly different from the *Kcnq2* mRNA level in naïve rats (n = 6, Fig. 7A). Thirty days after injury, the *Kcnq2* mRNA levels in ipsilateral DRG of neuropathic animals decreased to 0.09 ± 0.03 (n = 8) as compared with values of 0.31 ± 0.05 (n = 6) in naive and 0.23 ± 0.05 (n = 7) in sham controls (*P* < .05). Additionally there was a tendency of *Kcnq2* mRNA to decrease in the contralateral DRG of PSNL rats as well, but it did not reach significance (*P* = .09). Expression levels of *U6* did not differ between any of the experimental groups (not shown).

*Kcnq* gene expression is negatively regulated by the transcriptional repressor REST. Thus, it was shown that a functional RE1 site within *Kcnq2* can physically interact with REST, which results in suppression of *Kcnq2* expression [32]. Accordingly, overexpression of REST in DRG neurons led to a prominent decrease in *Kcnq2* mRNA level, Kv7.2 protein level, and M current density and resulted in hyperexcitable neurons [32]. We therefore tested whether *Rest* expression is changed after nerve injury. Relative *Rest* mRNA levels in neuropathic animals 15 days postinjury displayed large variability, but by 30 days *Rest* mRNA levels in ipsilateral DRG of neuropathic rats reached the level of 0.02 ± 0.004 (n = 8), which was significantly higher than the levels in naïve rats or sham controls (0.004 ± 0.001, n = 7 and 0.008 ± 0.002, n = 6; *P* < .05) (Fig. 7B). In line with the case of *Kcnq2,* there was a tendency for *Rest* mRNA to increase in the contralateral DRG of PSNL rats as well, but it did not reach significance (*P* = .13). In summary, there was a general tendency for *Kcnq2* mRNA levels to decrease and for *Rest* mRNA to increase in both ipsilateral and contralateral L4/L5 DRG after the PSNL injury. However, only the ipsilateral DRG showed significant changes in the expression of these genes.

**Fig. 8:**
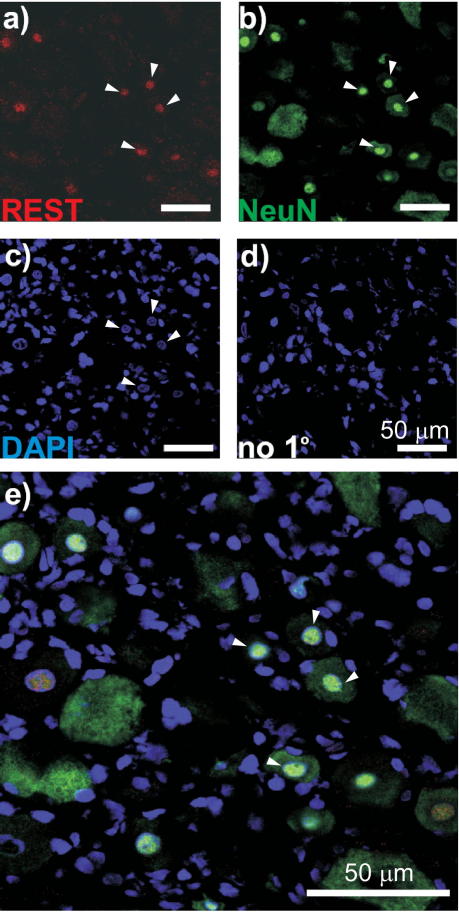
Expression of REST in DRG neurons. Confocal micrographs for immunostaining with antibodies against REST (a); the neuronal nuclei marker NeuN (b); DAPI nuclear staining (c); no-primary control (d) and co-staining with REST, NeuN, and DAPI (e). Scale bars = 50 μm.

**Fig. 9:**
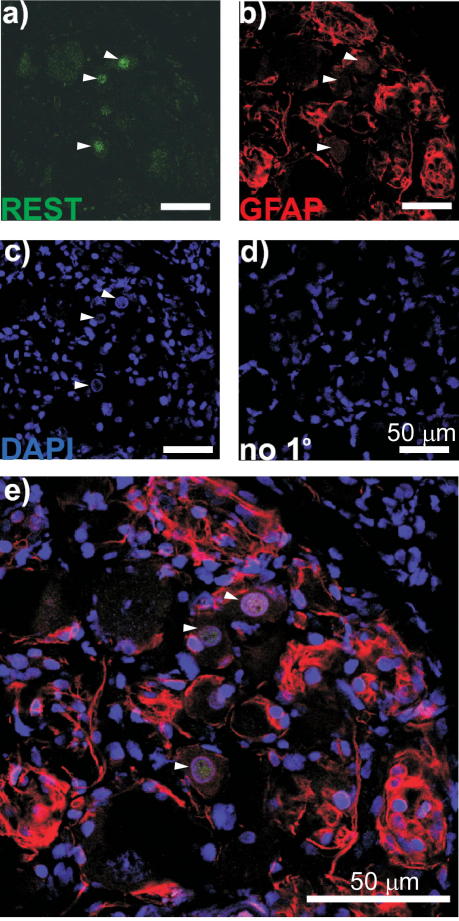
Lack of REST expression in satellite glial cells (SGC). Confocal micrographs for immunostaining with antibodies against REST (a); the glial cell marker GFAP (b); DAPI staining of glial and neuronal nuclei (c); no-primary control (d) and co-staining with REST, GFAP, and DAPI (e). Scale bars = 50 μm.

**Fig. 10:**
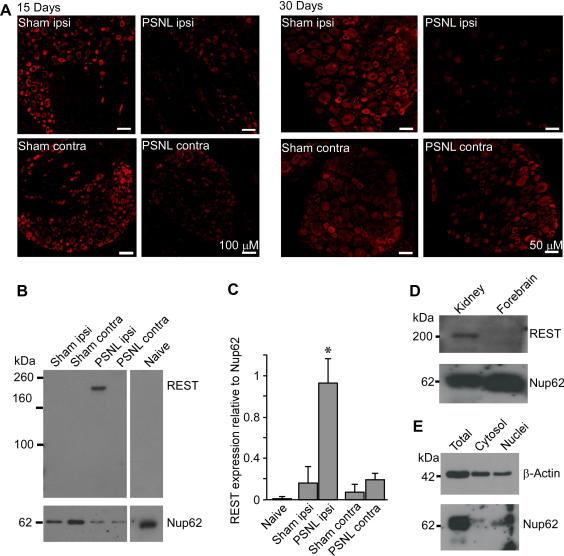
Reciprocal changes in the expression of Kv7.2 and REST proteins after peripheral nerve injury. (A) Confocal micrographs of DRG sections stained with anti-Kv7.2 antibody from 15 days (left 4 panels) and 30 days (right 4 panels) postsurgery PSNL or control (sham) animals. Scale bar = 50 μM. (B) Western blots of the nuclear-enriched fraction of DRG lysates from sham, PSNL (30 days after injury), and naive animals using anti-REST (REST) antibody and anti-Nup 62 (Nup 62). (C) Protein levels were quantified and REST levels normalised to the levels of Nup 62. Differences were analysed using one-way analysis of variance Holm–Sidak multiple comparison test, (^∗^*P* < .05, naïve n = 3, sham and PSNL n = 8). (D) Western blots of proteins extracted from kidney and forebrain probed with anti-REST and anti-Nup 62 antibody. (E) Western blot of proteins from total, cytosolic, and nuclear fractions from DRG probed with β-actin and anti-Nup 62 antibodies.

To test whether the expression of *Rest* and *Kcnq2* mRNA is mirrored by the appropriate changes in protein levels, we performed immunohistochemical analysis. First, we characterized expression of REST protein in DRG. Using antibodies against REST, neuronal nuclei marker NeuN (Fig. 8), or satellite glial cell (SGC) marker GFAP (Fig. 9), we found low but distinct REST immunoreactivity within the DRG neuron nuclei and almost no expression in SGC. We then evaluated changes in Kv7.2 and REST expression after the PSNL using immunohistochemistry and Western blot. Consistent with our RT-PCR data, Kv7.2 immunoreactivity was markedly reduced in the ipsilateral L4 DRG of neuropathic rats. Reduced staining for Kv7.2 in the ipsilateral DRG can be seen even 15 days after surgery (compared with sham controls), and by 30 days the reduction was prominent (Fig. 10A). Changes in Kv7.2 staining at the contralateral DRG of the PSNL animals were not dramatic. Similarly, REST protein abundance also increased dramatically in the nuclear-enriched fraction of the whole-DRG lysates from ipsilateral L4/L5 of PSNL rats as compared with samples from naive or sham controls (Fig. 10B and C). In accord with reported low expression of REST in the CNS (particularly in forebrain [34]), REST immunoreactivity was detected within kidney tissue but not in forebrain (Fig. 10D). Successful fractionation of the DRG lysates into nuclear and cytosolic fractions was assessed by β-actin (predominantly cytosolic protein) and Nup62 (nuclear membrane protein) immunoreactivity (Fig. 10E).

### 3.3 Local injection of M channel opener to injury site caused a decrease in thermal hyperalgesia

Recent reports demonstrated that inhibition of M channel activity at the peripheral nerve terminals of the sciatic nerve is, on its own, sufficient to cause overexcitability and pain [25,26]. Moreover, M current inhibition by such inflammatory mediators as bradykinin [26] or proteases [25] contributes to DRG excitability and hyperalgesia. Importantly, M channel opener retigabine reversed bradykinin-induced M channel inhibition (when applied to dissociated DRG neurons) and alleviated bradykinin-induced hyperalgesia (when injected into the hind paw) [26]. In addition, it was shown that systemic administration of retigabine alleviated neuropathic pain [12], whereas direct retigabine application to axotomised afferents reduced their excitability [40].

**Fig. 11:**
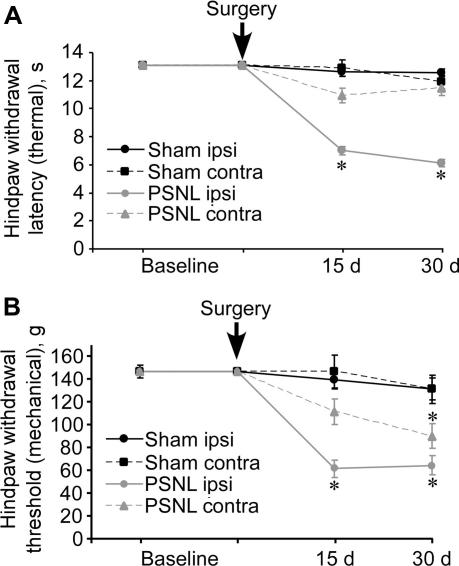
Behavioural analysis of PSNL animals. (A) Thermal hyperalgesia measured as the hind paw withdrawal latency (see Section 2) at baseline and 15 and 30 days postsurgery of the ipsilateral and contralateral paws form control and PSNL rats; n ⩾ 11; Kruskal–Wallis one-way analysis of variance on ranks with Dunn’s multiple comparison test (^∗^*P* ⩽ .05). (B) Mechanical hyperalgesia as the hind paw withdrawal threshold (see Section 2) at baseline and 15 and 30 days postsurgery of the ipsilateral and contralateral paws form control and PSNL rats; n ⩾ 11; Kruskal–Wallis one-way analysis of variance on ranks with Dunn’s multiple comparison test (^∗^*P* < .05).

According to the data presented here, M channel abundance within lumbar DRG decreases significantly after PSNL. Therefore, we tested whether some of the neuropathic pain symptoms could be attenuated by peripheral augmentation of M channel activity. First, we evaluated the development of thermal (Fig. 11A) and mechanical (Fig. 11B) hyperalgesia following the PSNL at time intervals matching our previous studies. PSNL rats exhibited unilateral thermal hyperalgesia at the ipsilateral hind paw and also exhibited mechanical hyperalgesia, which was unilateral 15 days after injury and became bilateral by 30 days (Fig. 11A and B). The original report of the PSNL model [43] described mirror thermal and mechanical hyperalgesia in the contralateral hind paw; however, the lack or delayed bilaterality has been reported by others [4,22]. In the present study we observed consistent mechanical and thermal hyperalgesia on the ipsilateral paw and delayed mechanical hyperalgesia on the contralateral paw.

**Fig. 12:**
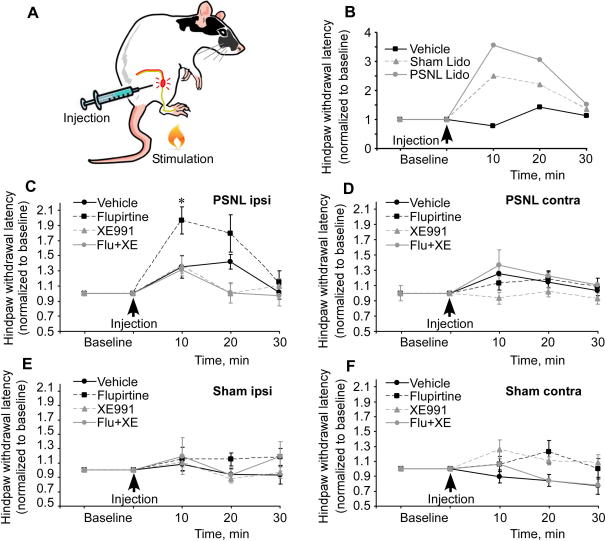
Perisciatic nerve injection of M channel opener alleviated thermal hyperalgesia produced by PSNL. (A) Schematic representation of the perisciatic nerve injection; injured nerve with neuroma is depicted in red. (B) Perisciatic nerve injection of lidocaine caused a decrease in thermal hyperalgesia in PSNL animals, confirming accuracy of sciatic nerve injection method. Neuroma site of PSNL rats was injected with 200 μL of 0.65% lidocaine solution, which caused a decrease in thermal hyperalgesia as compared with vehicle. (C–F) Hind paw withdrawal latency measured in PSNL (C and D) and sham (E and F) rats for ipsilateral (C and E) and contralateral (D and F) paws in response to injections of vehicle, flupirtine (200 μL, 10 nmol/site), XE991 (200 μL, 2 nmol/site) or flupirtine and XE991. All experiments in B through F were performed 30 days after the surgery; site of injection is depicted in (A). ^∗^*P* < .05; one-way analysis of variance with Dunn’s multiple comparison procedure (n ⩾ 4).

To probe whether pharmacological M channel augmentation within the injured nerve fibres would alleviate PSNL-induced hyperalgesia, we looked for a protocol that would allow manipulation of M channel activity at the site of neuroma. Hind paw injections of M channel drugs would not be the best approach because in PSNL ligated fibres are cut below the ligature site and degenerate. On the other hand, it has been suggested that the neuropathic hyperalgesia after the peripheral nerve lesion is maintained by central sensitization within the spinal cord resulting from the constant input from the ectopic afferent discharge from the site of peripheral nerve injury [16,44,47]. This central sensitization amplifies the input from uninjured fibres resulting in hyperalgesia. Accordingly, local application of low doses of lidocaine specifically to injured fibres in the SNL model of neuropathic pain alleviated hypersensitivity of intact fibres [47]. We reasoned that pharmacological restoration of M channel function by injecting M channel opener directly into the perisciatic nerve (PSN) space surrounding neuroma (Fig. 12A) may dampen the ectopic firing from neuroma and alleviate the neuropathic hyperalgesia by way of reducing spinal sensitization. To test whether we indeed can deliver drugs directly to the injured sciatic nerve, we injected lidocaine (200 μL of 0.65% v/v) directly into neuroma, which produced a transient (approximately 2 minutes) period of paralysis followed by a marked increase in thermal latency of the ipsilateral hind paw withdrawal in PSNL rats (Fig. 12B), confirming the accuracy of PSN injection. We then injected the M channel opener flupirtine (chemical analogue of retigabine) to test its effects on neuropathic hyperalgesia. PSN injection of flupirtine (200 μL, 10 nmole/site) into the neuroma site of rats 30 days after injury significantly increased hind paw withdrawal latency in the ipsilateral paw of PSNL animals, whereas such injections were without significant effect when injected contralaterally into the muscles surrounding intact sciatic nerves in sham controls (Fig. 12C through F). Importantly, the effect of flupirtine was completely blocked by co-injection of XE991 (200 μL, 2 nmole/site; Fig. 12D). Consistent with our hypothesis, XE991 injected alone did not affect paw withdrawal latency in either of the experimental conditions. In an uninjured paw (contralateral PSNL, sham and naive rats), peripheral nerve endings within the plantar surface of the paw are the sole source of excitation in response to stimuli applied to the plantar paw, an area that was not affected by the injection (Fig. 12A). In the injured paw, the neuroma site provides an additional source of activity, but because of the decreased level of *Kcnq2* expression, the tonic M channel activity must be very low and additional M channel inhibition has no further effect. However, by activating the M channels that are present, the M channel opener reinstates M current levels and thus reduces thermal hyperalgesia to almost basal level (compare Fig. 11A and Fig. 12C). In summary, the behavioural tests supported our hypothesis that M channel activity within the peripheral nerves controls the fibre excitability and that decreased M current may contribute to hyperalgesia; furthermore, we demonstrated that pharmacological targeting of peripheral M channels is efficacious against neuropathic hyperalgesia.

## 4 Discussion

We investigated functional expression in sensory neurons of the *Kcnq2* gene encoding the K^+^ channel subunit Kv7.2, which forms membrane potential stabilizing M channels. We found that Kv7.2 is expressed in a large number of small nociceptive neurons, many of which also expressed the nociceptive markers IB4 and TRPV1, whereas only a small number of larger, NF200-positive neurons were Kv7.2-positive. Although precise measurement of colocalization of immunoreactivities is difficult and there is always some degree of colocalization by chance, our conclusion that Kv7.2 is predominantly expressed in small nociceptive neurons is supported by the size distribution of the Kv7.2-positive neurons (Fig. 1B). Furthermore, previous studies have reported that approximately 30% of small sensory neurons are IB4 positive [3] and approximately 50% of small to medium DRG neurons are TRPV1positive [17]. These proportions are similar to that for the percentage of Kv7.2-positive neurons that are IB4 or TRPV1 positive. We hypothesize that the higher levels of M channel expression in nociceptors as compared with other sensory neurons may underlie the higher threshold for action potential firing of nociceptors [31]. M channel activity provides an intrinsic voltage clamp mechanism allowing neurons to stabilize the resting membrane potential at voltages near the M channel activation threshold [7]. Therefore, a high level of functional M channel expression results in a neuron with reduced excitability (as was directly shown for sympathetic neurons [21]).

M channel inhibition in nociceptive neurons was shown to increase their excitability and was sufficient to induce a moderate level of pain [25,26]. We therefore hypothesized that long-term downregulation of the M channels may contribute to hyperexcitability of sensory fibres in neuropathic injury. Indeed, we observed strong downregulation of Kv7.2 in the ipsilateral DRG of the rats with PSNL. This downregulation correlated with an increase of the mRNA and protein levels of REST, which negatively regulates *Kcnq* gene expression (eg, transient overexpression of REST in DRG suppressed *Kcnq2* expression, reduced M current density, and produced highly excitable nociceptive neurons [32]). Importantly, other *Kcnq* genes expressed in DRG, *Kcnq3* and *Kcnq5*, can also be suppressed by REST [32]; therefore, stimulation of REST expression in neuropathic neurons should result in general downregulation of all M channels. We further hypothesize that REST expression in sensory neurons is induced by trauma and/or inflammation (as suggested in Mucha et al. [32]) accompanying the onset of neuropathic injury. Indeed, increase of REST expression in sensory neurons days after the neuropathic injury has been reported [49], and in our study we saw a tendency for *Rest* upregulation at 15 days postinjury and further significant upregulation at 30 days (when significant decrease of *Kcnq2* expression was observed). Importantly, the efficiency of transcriptional suppression of target genes by REST depends on the REST affinity of the individual RE1 sites and from REST concentration [33]. Therefore, we suggest that gradual increase of *Rest* expression after nerve injury can cause a successive knock-down of its target genes in accordance with the strength of interaction between REST and its discrete binding sites. Noteworthy is the fact that neuropathic pain-associated suppression of several REST target genes has been demonstrated; these include genes encoding the K^+^ channels Kv4.3 [50] and Kv3.4 [8], the Na^+^ channel Nav1.8 [49], and the μ-opioid receptor [49,53]. Future research is needed to correlate the timing of the changes in REST target gene expression after the nerve injury with the affinity of their REST binding sites.

Changes of neuronal mRNA and protein levels measured using whole DRG lysates may be the result of neuronal death or proliferation of SGC, indeed both effects would not be surprising after an injury. However, because within DRG, both *Kcnq2* and *Rest* are predominantly expressed in neurons (Figs. 1C, 8, and 9), a significant decrease in the neuron/glia ratio would result in a simultaneous decrease of both gene products, which was not the case as *Rest* mRNA and protein levels increased while those of *Kcnq2* decreased. In addition, we performed retrograde staining of cell bodies of damaged fibres at the time of PSNL surgery (data not shown) that demonstrated that between 15 and 30 days after PSNL there was no noticeable neuronal death. SGC proliferation in DRG does occur after nerve injury; however, several studies reported that there may be 2 waves of such proliferation. The first wave starts immediately after injury with glial cell number returning to the baseline by the end of the first week (this most likely is an inflammatory process [20]); the second wave is characterized by formation of “onion bulbs”—DRG neuron somata wrapped in multiple SGC layers [45], however, this process is not apparent until the 5th week after injury and was not seen in any DRG sections in this study. Because our experiments were performed at 15 and 30 days after injury, it is possible that this period falls between 2 waves of SGC proliferation.

Neuronal population of L4 and L5 DRG in PSNL animals is comprised of axotomised and intact neurons. Although we did not specifically test whether these neuronal subpopulations responded differently in terms of *Kcnq2* and *Rest* expression, our data suggest that there is rather a global effect of partial axotomy on the expression of these genes in DRG (eg, Fig. 10A). In addition, our data suggest that hyperalgesia in PSNL animals appears sooner than the *Kcnq2* downregulation occurs; thus significant downregulation of *Kcnq2* was only detected at 30 days postinjury (Fig. 7A), whereas the hyperalgesia was already developed by 15 days (Fig. 11). Although immunohistochemistry (Fig. 10A) suggests that there is some downregulation of Kv7.2 in DRG of PSNL animals at 15 days postinjury (which may reflect poor sensitivity of the RT-PCR analysis), the fact that hyperalgesia is already fully developed by 15 days whereas *Kcnq2* downregulation is not suggests that the *Kcnq2* downregulation is a delayed feature of nerve injury that is developed in response to the initial hyperexcitability produced by other factors. It is conceivable that the initial hyperexcitability of peripheral fibres after the injury is driven by more acute factors such as trauma and inflammation (to which acute inflammatory inhibition of M channel activity most likely contributes [25,26]); therefore, the transcriptional mechanism described here may have a role in the maintenance of pain rather than in its initiation. The injury-induced inflammation is likely to affect both axotomised and intact fibres within the affected nerve, and we recently presented evidence that inflammatory excitation triggers *Rest* expression in DRG neurons, which in turn causes downregulation of *Kcnq2* [32]. Thus, it is logical to expect some global decrease in REST target gene expression after partial axotomy, although the severed fibres will most likely be the most affected. Future experiments will test whether this hypothesis is correct.

To test the role of M channel regulation in neuropathic pain in vivo, we used a behavioural paradigm. In accord with previous findings suggesting that augmentation of M current in sensory fibres has an antinociceptive effect [6,18,24,40], we demonstrated that thermal hyperalgesia produced by neuropathic injury is alleviated by PSN injection of the M channel opener flupirtine, an effect that was completely blocked by the M channel blocker XE991. Interestingly, PSN injections of flupirtine did not affect thermal withdrawal latencies in uninjured paws. Although the site of injection in our experiments did not encompass the site of stimulation (Fig. 12A), one can expect that PSN injection of M channel opener could hyperpolarize the area of the nerve and to some extent block action potential propagation. It was suggested that the membrane potential in sympathetic and sensory neurons can be set by a dynamic equilibrium of 2 opposing currents, depolarizing I*_h_* (hyperpolarization-activated current conducted by HCN channels) and hyperpolarizing I*_M_*; with I*_h_* being activated at the potentials negative to −50 mV and I*_M_* activating at potentials positive to −60 mV [1,23]. Thus, we speculate that the lack of any significant effect of M channel augmentation on uninjured paws may result from the offsetting action of I*_h_*. In contrast, when M channel abundance is decreased and the fibre is tonically depolarised, activation of residual M channels results in reversion of depolarization back to the level at which I*_h_* starts coming into play, which results in a reset of an overexcitable neuron into a low-excitability state manifested in the increase of hind paw withdrawal latency (Fig. 12C).

There are reports of sympathetic sprouting and proliferation after PSNL injury resulting in sympathetically maintained pain [46]. Further, it was suggested that the central analgesic action of flupirtine is via alterations in descending sympathetic tone [48]. Although the injected dose of flupirtine was too low to cause a significant central effect, it cannot be excluded that alterations in Kv7.2 expression within sympathetic postganglionic neurons and/or alterations in sympathetic tone contributed to the analgesic effect of flupirtine reported here.

In conclusion, we have identified a novel transcriptional mechanism that may contribute to the neuropathic overexcitability of peripheral sensory fibres: the downregulation of the *Kcnq2* gene encoding the M channel subunit Kv7.2 by REST. Our data identify M channels and REST as peripheral targets for treatment of pain.

## Figures and Tables

**Table 1:** Primary and secondary antibody information.

Primary antibody	Dilution	Reference/supplier	Secondary antibody	Supplier
Rabbit anti-Kv7.2	1:2000	Roche et al. [39]	Donkey anti-rabbit Alexa 555/488	Invitrogen
Rabbit anti-REST	1:500	Santa Cruz	Donkey anti-rabbit Alexa 555	Invitrogen
Mouse anti-NF200	1:1000	Sigma	Donkey anti-mouse Alexa 488	Invitrogen
Biotinylated IB4	1:1000	Sigma	Streptavidin-conjugated Alexa 488	Invitrogen
Guineapig anti-TRPV1	1:1000	Neuromics	Goat anti-guinea pig Alexa 488	Invitrogen
Cy3-conjugated anti-GFAP	1:500	Sigma	N/A	N/A
